# Skin Vaccination with Ebola Virus Glycoprotein Using a Polyphosphazene-Based Microneedle Patch Protects Mice against Lethal Challenge

**DOI:** 10.3390/jfb14010016

**Published:** 2022-12-27

**Authors:** Andrey Romanyuk, Ruixue Wang, Alexander Marin, Benjamin M. Janus, Eric I. Felner, Dengning Xia, Yenny Goez-Gazi, Kendra J. Alfson, Abdul S. Yunus, Eric A. Toth, Gilad Ofek, Ricardo Carrion, Mark R. Prausnitz, Thomas R. Fuerst, Alexander K. Andrianov

**Affiliations:** 1 School of Chemical and Biomolecular Engineering, Georgia Institute of Technology, Atlanta, GA 30332, USA; 2 Institute for Bioscience and Biotechnology Research, University of Maryland, Rockville, MD 20850, USA; 3 Department of Pediatrics, Division of Endocrinology, Emory University School of Medicine, Atlanta, GA 30322, USA; 4 School of Pharmaceutical Sciences (Shenzhen), Sun Yat-sen University, Shenzhen 518107, China; 5 Texas Biomedical Research Institute, San Antonio, TX 78227, USA; 6 Department of Cell Biology and Molecular Genetics, University of Maryland, College Park, MD 20742, USA

**Keywords:** microneedle patch, polyphosphazene, immunoadjuvant, Ebola vaccine, intradermal immunization, supramolecular assembly, lethal challenge

## Abstract

Ebolavirus (EBOV) infection in humans is a severe and often fatal disease, which demands effective interventional strategies for its prevention and treatment. The available vaccines, which are authorized under exceptional circumstances, use viral vector platforms and have serious disadvantages, such as difficulties in adapting to new virus variants, reliance on cold chain supply networks, and administration by hypodermic injection. Microneedle (MN) patches, which are made of an array of micron-scale, solid needles that painlessly penetrate into the upper layers of the skin and dissolve to deliver vaccines intradermally, simplify vaccination and can thereby increase vaccine access, especially in resource-constrained or emergency settings. The present study describes a novel MN technology, which combines EBOV glycoprotein (GP) antigen with a polyphosphazene-based immunoadjuvant and vaccine delivery system (poly[di(carboxylatophenoxy)phosphazene], PCPP). The protein-stabilizing effect of PCPP in the microfabrication process enabled preparation of a dissolvable EBOV GP MN patch vaccine with superior antigenicity compared to a non-polyphosphazene polymer-based analog. Intradermal immunization of mice with polyphosphazene-based MN patches induced strong, long-lasting antibody responses against EBOV GP, which was comparable to intramuscular injection. Moreover, mice vaccinated with the MN patches were completely protected against a lethal challenge using mouse-adapted EBOV and had no histologic lesions associated with ebolavirus disease.

## 1. Introduction

Ebolavirus (EBOV) infection in humans causes severe hemorrhagic fevers with high mortality rates as recently shown by the 2013–2016 outbreak in West Africa that caused more than 28,600 human infections and over 11,300 deaths [1]. More recently, the Ministry of Health of the Democratic Republic of the Congo (DRC) declared an outbreak of Ebola Virus Disease (EVD), the fourteenth EVD outbreak in DRC since 2018 [2,3]. The high mortality rate and lack of effective interventional strategies for prevention or treatment of infection highlight the importance for developing a safe and effective EBOV vaccine to address this public health need. The currently licensed vaccines in the United States and Europe, under exceptional circumstances, all use viral vector platforms expressing the EBOV envelope glycoprotein (GP) antigen to stimulate an immune response [4,5,6]. GP is located on the surface of the virion and mediates attachment, fusion, and entry into target cells, and serves as the main target of a neutralizing antibody response [7].

However, these viral vaccine vectors have general disadvantages, such as difficulties in manufacturing and adapting to new virus variants, reliance on cold chain supply networks, and injection by hypodermic needle. Injection by trained health care providers is often difficult to perform in developing countries with limited infrastructure, particularly in situations in which the vaccine needs to be rapidly deployed in remote locations. In view of these limitations, there is a need for an improved EBOV vaccine that has simplified logistics for rapid vaccination coverage, especially in environments with limited health care infrastructure.

Microneedle (MN) patches have been developed for administration of a number of vaccines in pre-clinical studies [8,9,10,11,12,13,14,15,16], and have been the subject of phase 1 clinical trials of influenza vaccination [17,18]. These patches are made of an array of micron-scale, solid needles that painlessly penetrate into the upper layers of the skin [19,20,21,22]. When pressed to the skin, the MNs dissolve within minutes to release encapsulated vaccine and adjuvant without the need for hypodermic needles or injection. This novel vaccine technology offers a number of advantages over more-conventional delivery approaches such as intramuscular (IM) injection by hypodermic needles with respect to vaccine stability, reduced pain during administration, storage conditions, ease of use, and elimination of biohazardous sharps waste [12,20,23,24,25]. By administering vaccine to the skin, MN patches can enhance vaccine immunogenicity by targeting epidermal Langerhans cells, dermal dendritic cells, and lymphatic drainage from skin [26,27,28]. 

Over the past two decades, the polyphosphazene-based adjuvant system composed of water-soluble synthetic macromolecules with a biodegradable backbone has been explored with multiple vaccine antigens [29,30,31]. These hybrid organic-inorganic macromolecules have been proven to display a potent immunopotentiating effect in vivo, which manifests in improved magnitude, quality, onset, and duration of immune responses to the antigen, and underlying vaccine dose sparing capacity [29,32]. The lead polyphosphazene adjuvant, poly[di(carboxylatophenoxy)phosphazene] (PCPP), has been advanced into clinical trials, and PCPP-adjuvanted vaccines are reported to be safe and immunogenic in humans [33,34,35]. The ability of PCPP to spontaneously self-assemble with vaccine antigens in aqueous solutions results in its ability to stabilize proteins upon drying or thermal treatment [36,37,38,39], which makes it an attractive candidate for applications involving dehydration of vaccine formulations. The material properties of PCPP in a solid state are defined by its polymer nature and can contribute to the mechanical strength of MNs [40].

Building off our prior studies of MN patch vaccination with EBOV vaccine [24], the present study describes a potential Ebola vaccine composed of EBOV GP formulated with a polyphosphazene-based adjuvant system to improve vaccine immunogenicity and stability, and administered by an MN patch designed to simplify vaccination and thereby improve vaccination coverage. The present results show that a PCPP-based, dissolvable, EBOV GP MN patch vaccine can be made while maintaining antigenicity of EBOV GP, and that immunization of mice by MN patches induced high-level, long-lasting antibody responses against EBOV GP that was comparable to IM injection. Moreover, mice vaccinated with the MN patches were completely protected against lethal mouse-adapted EBOV challenge and had no histologic lesions associated with ebolavirus disease.

## 2. Materials and Methods

### 2.1. Expression and Purification of EBOLA Virus Glycoprotein Antigens and Antibodies

The Ebola Zaire GP ectodomain with deleted mucin-like and transmembrane domains (EBOV GPΔMuc) was expressed and purified as previously described [41]. Briefly, a codon-optimized EBOV GPΔMuc construct corresponding to the sequence of strain Mayinga-76 GP and containing C-terminal polyhistidine and Strep-II tags was used. It was transiently transfected into HEK293S GNTI-/- cells using 293fectin transfection reagent according to manufacturer’s guidelines (Thermo Fisher Scientific, Waltham, MA, USA). Furin protease was co-transfected at a 30% ratio to ensure proper cleavage of GP. Secreted EBOV GPΔMuc protein was harvested from supernatants and purified using cOmplete His-Tag Purification Resin (Roche CustomBiotech, Indiananapolis, IN, USA) followed by Strep-Tactin Sepharose Resin (IBA Lifesciences, Göttingen, Germany) purification. The GP protein was further purified by size exclusion chromatography using a Superdex 200 HiLoad 16/600 column in 150 mM NaCl, 2.5 mM Tris 7.5 and 0.002% (*w*/*v*) NaN_3_. Following purification, the protein was dialyzed into 50 mM sodium phosphate buffer (pH 7.4) and concentrated to 5 mg/mL. 

The FVM04 and FVM09 antibody IgGs were prepared as previously described [41]. Briefly, antibody heavy and light chain expression plasmids were transiently co-transfected into HEK-293F cells (ATCC, Manassas, VA, USA) using 293Fectin and grown in Freestyle media as per manufacturer’s guidelines (Thermo Fisher Scientific). The supernatants were harvested after 3–4 days and the IgGs were purified using Protein A Resin (Roche CustomBiotech) and eluted with IgG Elution Buffer (Thermo Fisher Scientific) followed by immediate neutralization with Tris Base pH 9.0.

### 2.2. Polyphosphazene Formulations

PCPP (800,000 g/mol) was synthesized and characterized as described previously [42], dialyzed against deionized water to remove salts, and lyophilized. Dynamic light scattering (DLS) characterization of PCPP formulations was carried out using Malvern Zetasizer Nano series instrument (Malvern Instruments, Worcestershire, UK). Asymmetric flow field flow fractionation (AF4) analysis was conducted using a Postnova AF2000 MT instrument (Postnova Analytics, Landsberg, Germany). 

### 2.3. Fabrication of Microneedle Patches

Fabrication of MN patches was carried out by a two-step solvent-casting process [43,44,45,46]. A first casting solution was prepared to contain 3.75 mg/mL EBOV GPΔMuc antigen, 12.5 mg/mL PCPP, and 100 mg/mL sucrose (Sigma-Aldrich, St. Louis, MO, USA) in 100 mM phosphate buffer pH 7.4. This solution was cast onto a polydimethylsiloxane (PDMS) micromold (5 µL volume) having the inverse shape of an MN patch and exposed to vacuum to facilitate filling the MN cavities. Excess casting solution was removed from the mold surface and samples were dried in ambient air (20–25 °C, 30–60% relative humidity) for 20 min. 

Then, a second casting solution (25% (*w*/*v*) gelatin (Sigma-Aldrich) and 20% (*w*/*v*) sucrose) in 100 mM phosphate buffer (pH 7.4) was cast onto the mold to form the MN patch backing. Patches were dried at ambient temperature overnight and for 24 h under vacuum at 4 °C in a desiccator, demolded, and stored at room temperature in a sealed aluminum pouch with desiccant. In some patches, sulforhodamine B dye (Sigma-Aldrich) was added to facilitate MN imaging. For comparison purposes, PCPP-free antigen-loaded MN patches were also fabricated. In these patches, PCPP was substituted with medium viscosity carboxymethylcellulose (CMC, Sigma-Aldrich, St. Louis, MO, USA), which is a commonly used material in MN patches [43,47,48].

MN patch mechanical strength was assessed by applying MN patches to pig skin ex vivo. After removing the MN patches from the skin, gentian violet dye (Humco, Austin, TX, USA) was applied to the skin for 10 min and then wiped off with isopropyl alcohol wipes. The dye selectively stained sites of puncture in the skin by the MNs.

### 2.4. Analysis of Microneedle Patches for Antigenicity

Antigen content in MN patches was evaluated using enzyme-linked immunoassay (ELISA). A 96-well plate was coated overnight at 4 °C with 100 µL of 1 µg/mL of FVM04 monoclonal antibody in phosphate-buffered saline (PBS). The coating solution was removed from the plate, which was then washed with PBS, blocked by addition of 300 µL/well of 2% (*w*/*v*) bovine serum albumin (Sigma-Aldrich)/0.05% (*w*/*v*) Tween-20 (Sigma-Aldrich) in PBS for 1 h at ambient temperature and then washed with 0.05% (*w*/*v*) Tween-20 in PBS. 

MN patches were dissolved in 1 mL of PBS. The resulting solution was diluted ten-fold with blocking buffer, and 100 µL of it was added to each well on the 96-well plate. The calibration curve was prepared using standard solutions containing antigen, sucrose, gelatin, and PCPP at a mass ratio of 1:187:187:4.7 (i.e., the same ratio as in MN patches) diluted in blocking buffer. The plate was incubated for 1 h at ambient temperature and washed as above. Then, 100 µL of peroxidase AffiniPure goat anti-human IgG (H+L, Jackson ImmunoResearch, West Grove, PA, at a 1:2500 dilution) was added to each well and incubated for 1 h at ambient temperature. The plate was washed as above, 100 µL of 3,3′,5,5′-tetramethylbenzidine, (TMB, MilliporeSigma, St. Louis, MO, USA) was added and incubated for 15 min at ambient temperature, and the reaction was stopped by adding 100 µL of 1 M sulfuric acid to each well. The absorbance of each well was read at 450 nm using a Multiskan Spectrum Reader (Thermo Fisher Scientific).

### 2.5. Animal Vaccination

Two-month-old female BALB/c mice (Charles River, Wilmington, MA, USA) were used. The mice were divided into three groups of six mice each to receive: (a) skin vaccination by application of MN patch that contained 13.4 µg EBOV GP vaccine with 62.5 µg PCPP adjuvant (MN-PCPP), which was estimated to deliver ~7 µg EBOV GP vaccine with ~31 µg PCPP adjuvant, based on an expected skin delivery efficiency of 50%; (b) IM injection of 50 µL PBS (pH 7.4) containing 7 µg EBOV GP vaccine with 31 µg PCPP adjuvant (IM-PCPP); and (c) IM injection of 50 µL PBS (pH 7.4) containing 7 µg EBOV GP vaccine with no adjuvant (IM-No adjuvant). 

IM injections were administered by hypodermic syringe in hind-limb muscles. For skin vaccination, a 2 cm × 2 cm area on the dorsal side of the mouse was shaved and treated with depilatory cream (Nair, Church & Dwight, Pittsburgh, PA, USA) two days prior to the immunization. MN patches were applied to the prepared site, firmly held in place for 1 min and then left on skin for 20 min. Mice received immunizations on Days 0 and 28. No side effects, such as inflammatory response or tissue injury in the vaccinated region of skin, were observed. Blood samples were collected from jugular vein on Days 0, 14, 28, 42, 56 and 180, separated into serum using a capillary tube (Fisher Scientific, Pittsburgh, PA, USA) and stored frozen at −20 °C until analysis. The study was conducted according to the guidelines of the Declaration of Helsinki and approved by the Institutional Animal Care and Use Committee (IACUC) of Georgia Institute of Technology (protocol number: A100427; approval date: 1 June 2021).

### 2.6. Evaluation of Anti-GP Antibody Responses

EBOV GP-specific antibody responses were evaluated by antigen-capture enzyme-linked immunosorbent assay (ELISA) using the immune sera as previously described [49]. In brief, 96-well plates (MaxiSorp, ThermoFisher Scientific) were coated with 5 µg/mL Galanthus Nivalis Lectin (Vector Laboratories, Burlingame, CA, USA) overnight at 4 °C. The following day, plates were washed with PBS containing 0.05% Tween 20 and then coated with 200 ng/well Zaire GPΔmuc antigen at 4 °C. Plates were washed 3 times after overnight incubation and blocked with Pierce Protein-Free Blocking Buffer (ThermoFisher Scientific) for 1 h at room temperature. Serum from individual mice was then added to the plates and tested in duplicate at 5-fold serial dilutions. The binding of Zaire GP-specific antibodies was detected by 1:5000 diluted horseradish peroxidase (HRP)-conjugated goat anti-mouse secondary antibody (Abcam, Cambridge, MA, USA), followed by incubation with 100 µL of TMB substrate (Bio-Rad Laboratories, Hercules, CA, USA) for color development. The absorbance was measured at 450 nm using SpectraMax M3 microplate reader (Molecular Devices, San Jose, CA, USA). The antibody endpoint titer was determined as the highest reciprocal dilution of serum that resulted an optical density (OD) reading 4 times the value of pre-immune sera.

### 2.7. Pseudoparticle Neutralization Assay

Neutralizing activity of sera from vaccinated mice was analyzed against pseudoparticles containing EBOV GP (EBOVpp) based on a murine leukemia virus (MLV) backbone as described in previous studies [50]. Three different strains of EBOVpp were generated, EBOV-Zaire (EBOVpp-Zaire), EBOV-Sudan (EBOVpp-Sudan), and EBOV-Bundibugyo (EBOVpp-Bundibugyo). The pseudoparticles were made by co-transfection of HEK293T cells with the MLV Gag-Pol packaging vector (phCMV-5349), luciferase reporter plasmid (pTG126), and the plasmids expressing EBOV GP. Pseudoparticles made in the absence of EBOV GP expressing plasmid were used as a negative control. For serum neutralization, Vero E6 cells (ATCC, Manassas, VA, USA) were seeded overnight (CO_2_ incubator) in 96-well plates at a density of 1 × 10^4^ per well. The following day, pseudoparticles were mixed with serially diluted heat-inactivated serum for 1 h at 37 °C, and then added in duplicate to pre-seeded Vero cells. After incubation at 37 °C for 5 h, the mixtures were replaced with fresh medium and the plates continued to incubate. After 72 h, 100 µL BrightGlo (Promega, Madison, WI) was added to each well and the luciferase activity was measured in relative light units (RLUs) using a FLUOstar Omega plate reader (BMG Labtech, Ortenberg, Germany). The percentage of neutralization was calculated as follows:

100% × [1 − EBOVPPRLU (pp + sera + cells)/EBOVppRLU control (control pp + cells)]

where EBOVPPRLU (pp + sera + cells) is the RLU detected in the presence of the indicated pseudovirus and EBOVppRLU control (control pp + cells) is the RLU detected in the presence of a pseudovirus lacking any GP. 

nAbs titers in mice sera were reported as 50% inhibitory dilution (ID50) values. Neutralization curves and ELISA binding curves were fitted by nonlinear regression in GraphPad Prism 7 (GraphPad Software, San Diego, CA, USA). Significance comparisons were calculated using Kruskal–Wallis with Dunn’s multiple comparison by GraphPad software (GraphPad Software, San Diego, CA, USA). 

### 2.8. Challenge Study and Histopathological Examination

On Day 210 post-vaccination, four groups of mice were challenged with 1000 plaque-forming units (pfu) of mouse-adapted EBOV (MA-EBOV) via intraperitoneal injection [24]: the first three groups were the same animals as those described above in the vaccination study (i.e., MN-PCPP, IM-PCPP, and IM-No adjuvant), and the fourth group was an unvaccinated control group. After the challenge, mice that succumbed to the challenge and mice surviving to Day 28 were recorded for each group of mice. Necropsies were conducted to collect liver and spleens for histopathological examination from the surviving mice and those that succumbed to the challenge. Liver and spleen tissues from individual mice were each fixed by immersion in 10% neutral-buffered formalin for a minimum of fourteen days, then trimmed, processed, and embedded in paraffin. Sections of the paraffin-embedded tissues were cut to 5 µm, and histology slides were stained with hematoxylin and eosin (H&E), and evaluated by a board-certified veterinary pathologist using a light microscope. The evaluation of liver tissue included necrosis, hepatocellular inflammation, intracytoplasmic inclusions, fibrin deposition, fatty change, increased immunoblasts, and infiltration of vessel walls; and spleen tissue included decrease lymphocytes, lymphocytosis, fibrin deposition, follicular hyperplasia, increased immunoblasts, congestion/hemorrhage, necrosis, and increased macrophages. A numerical severity score was assigned for each tissue that ranged from 0 (not present), 1 (minimal), 2 (mild), 3 (moderate), 4 (marked), and 5 (severe). Microscopic findings in liver and spleen from unscheduled-death mice were typical of acutely fatal EBOV. EBOV-related findings in the liver included hepatocellular necrosis, inflammation, fibrin, and intracytoplasmic inclusion bodies typical of EBOV inclusions. EBOV-related findings in the spleen included decreased lymphocytes with lymphocytolysis and fibrin deposition. When present, the deposition of fibrin was scant. Increased immunoblasts were noted in the spleen. Cause of death was considered to be EBOV-related. The study was conducted according to the guidelines of the Declaration of Helsinki and approved by the IACUC of Texas Biomedical Research Institute (protocol number: 1788MU; approval date: 18 August 2021).

## 3. Results and Discussion

This study was designed to evaluate vaccination using EBOV GPΔMuc antigen administered by MN patches. Studies first characterized the antigen and MN patches, and then assessed immunogenicity and protective efficacy against challenge in mice. 

### 3.1. Ebola Virus Glycoprotein Antigen

**Figure 1:**
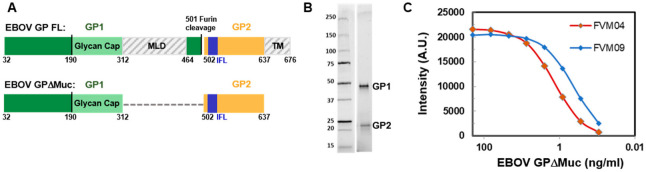
Ebola Virus GPΔMuc Antigen. (**A**) Shown are schematic representations of full-length EBOV GP (upper) and of the EBOV GPΔMuc antigen with deleted mucin-like and transmembrane domains (lower). (**B**) SDS PAGE analysis of purified of EBOV GPΔMuc antigen. (**C**) Antigenicity of EBOV GPΔMuc antigen was confirmed by binding of antibodies FVM04 and FVM09 that target the RBR and β17-18 loop on GP1, respectively.

EBOV GPΔMuc served as the antigen for our MN patch vaccination studies (Figure 1A) [41]. The protein was prepared by transient protein expression in HEK293S GNTI^-/-^ cells to yield a product with homogenous N-linked glycans. Secreted EBOV GPΔMuc protein was harvested from cell culture supernatants using appended C-terminal polyhistidine and Strep-II affinity tags for purification followed by size exclusion chromatography (Figure 1B). After purification, the antigen was dialyzed into 50 mM phosphate solution and concentrated to 5 mg/mL. Antigenic integrity of purified EBOV GPΔMuc was confirmed by ELISA using antibody FVM04 that targets the receptor binding region (RBR) on GP1 and antibody FVM09 that targets β17-18 loop on GP1 (Figure 1C) [51,52]. 

### 3.2. Formulation of Water-Soluble Supramolecular Complexes of Antigen and PCPP

**Figure 2:**
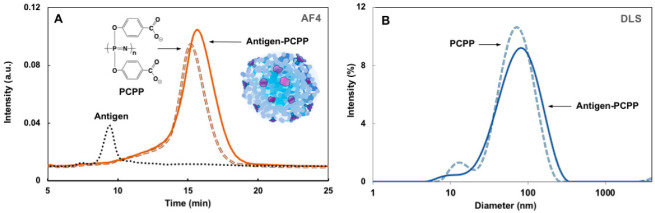
Characterization of antigen-PCPP complex. (**A**) Representative AF4 fractograms of antigen, PCPP, and antigen-PCPP complex. (**B**) Representative DLS profiles of PCPP and antigen-PCPP complex (0.05 mg/mL antigen and 0.25 mg/mL PCPP).

Formulation of EBOV GPΔMuc antigen with PCPP was carried out in aqueous solution at neutral pH and was monitored for potential self-assembly processes by the AF4 and DLS methods. No phase separation or aggregation could be visually detected upon mixing of stock solutions. Figure 2A shows AF4 fractograms of formulation components, i.e., PCPP and antigen, along with the antigen-PCPP mixture. The analysis allows size-based separation of analytes without imposing major limitations on their dimensions (up to micrometer size analytes can be characterized), minimizes interactions with stationary phase, and has been successfully employed for the analysis of polyphosphazene formulations [53,54,55]. 

The disappearance of the antigen peak (9.5 min) in the formulation and increase in the area of the peak corresponding to PCPP, along with its shift toward larger sizes (15–16 min), indicates antigen binding and formation of supramolecular complexes in the formulation. This conclusion is further confirmed by comparing the DLS profiles of PCPP and its formulation with the antigen, which shows a broader peak and greater z-average diameter (60 nm vs. 55 nm) for the antigen-PCPP system when compared to the polymer alone (Figure 2B). Taken together, these results demonstrate spontaneous self-assembly of antigen with PCPP in aqueous solution with the formation of supramolecular water-soluble complexes in formulations, suitable for incorporation into MN patches.

### 3.3. Microfabrication and Ex Vivo Testing of Microneedle Patches

The MN patches were fabricated by micromold casting to produce 100 solid, conical MNs in a 1 cm × 1 cm array with a loading of 13.4 ± 0.7 µg EBOV GPΔMuc and 62.5 ± 2.4 µg PCPP. The PCPP served three functions: stabilize the antigen during the MN patch fabrication process, adjuvant the immune response to the antigen, and provide mechanical strength to the MNs during their insertion into skin. The MNs also contained sucrose, which could further stabilize the antigen and facilitate rapid MN dissolution in the skin. The backing of the MN patch was made of gelatin, which provided mechanical strength, and sucrose for rapid dissolution. 

**Figure 3:**
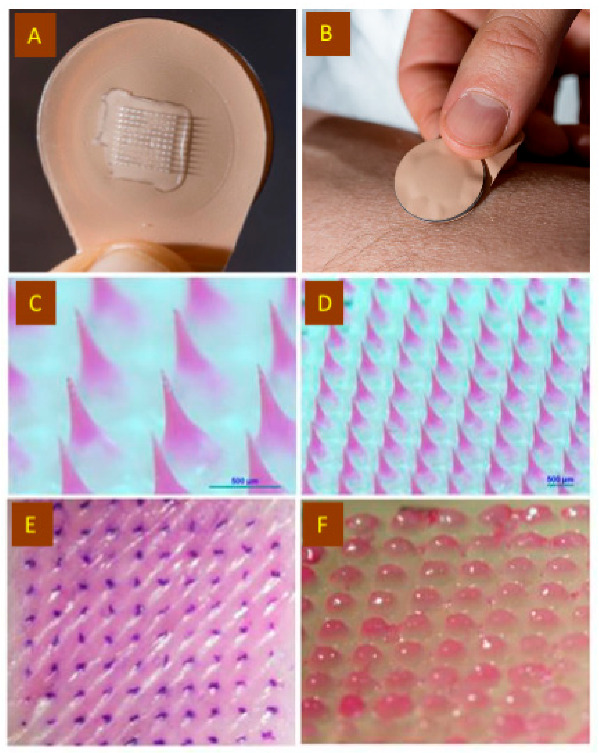
Microneedle patch application to skin. Representative photographic images of (**A**) a microneedle patch and (**B**) a microneedle patch being applied to the skin. Representative brightfield microscopy images of (**C**,**D**) microneedle arrays containing EBOV GPΔMuc antigen, PCPP, and sulforhodamine dye, (**E**) pig skin after ex vivo application of microneedle array and then applying a dye, (gentian violet) that stains sites of skin puncture and (**F**) microneedle patch after application to pig skin ex vivo, showing just the patch backing after microneedle separation and dissolution in skin.

The MN patches were designed to be small (Figure 3A) and simple to apply to the skin (Figure 3B). Figure 3C,D presents magnified views of the structure and layout of the MN patches containing fluorescent sulforhodamine loaded into the MN (to facilitate imaging), in addition to antigen and PCPP.

The dye-loaded patches were tested to confirm their ability to puncture into pig skin ex vivo. The patches were manually pressed to the skin by thumb without the need for specialized equipment or training. Inspection of the skin surface after removal of the patches showed an array of dye-stained spots corresponding to the sites of each MN insertion (Figure 3E). Imaging the patch after application to the skin confirmed the separation and dissolution of the MNs, as the used patch contained only the patch backing (Figure 3F). This dissolution of the MNs means that used patches produce no biohazardous sharps waste. 

### 3.4. Antigenicity of EBOV GPΔMuc in Microneedle Patches Containing PCPP 

**Figure 4:**
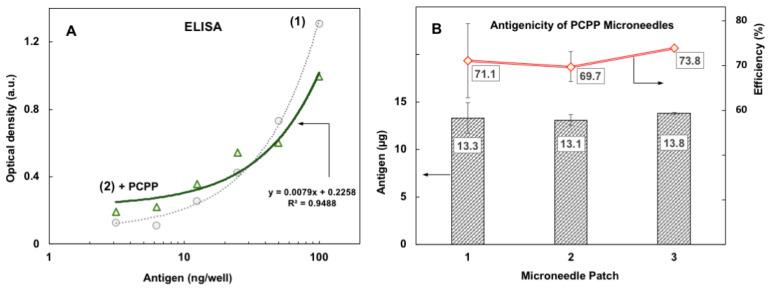
Antigenicity of EBOV GPΔMuc incorporated into microneedle patches. (**A**) Representative standard ELISA curves for EBOV GPΔMuc antigen formulations prepared in the presence (2) and absence (1) of PCPP (FVM04 monoclonal antibody; analyzed solutions also contained sucrose and gelatin to mimic microneedle patch formulation); (**B**) EBOV GPΔMuc antigen load (columns) and efficiency of antigen encapsulation (curve) of three PCPP microneedle patches, as determined by ELISA (analysis conducted using ELISA curve (1) of panel A; efficiency of encapsulation was determined as the ratio between experimentally detected antigen load after dissolution of microneedles and antigen load expected on the basis of microneedle formulation used for patch fabrication, expressed as percent; analysis conducted in triplicate; error bars represent standard deviation).

The MN patches containing EBOV GPΔMuc were dissolved and their antigenicity was determined by ELISA using the FVM04 monoclonal antibody. First, the effect of PCPP on the standard ELISA curves was evaluated to rule out potential interference of PCPP and other MN patch excipients with the analysis. The results revealed only minor effects of PCPP on the calibration curves (Figure 4A) and indicated that the quantitative analysis of EBOV GPΔMuc could also be performed in the presence of the MN patch formulation components, such as sucrose and gelatin. 

**Figure A1:**
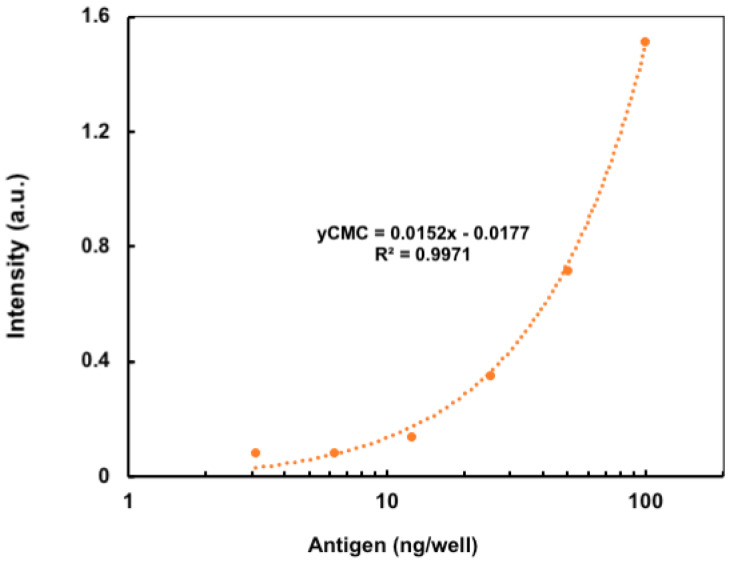
Standard ELISA curve for antigen formulations prepared in the presence of CMC (FVM04 monoclonal antibody); analyzed solutions also contained sucrose and gelatin to mimic microfabrication formulations.

Furthermore, the analysis of the MN patches formulated with PCPP-antigen complexes demonstrated that 71.5 ± 3.9% of EBOV GPΔMuc used in the MN patch fabrication process retained its antigenicity (Figure 4B). This is in contrast with the MN patches formulated with CMC instead of PCPP. The antigenicity of the CMC MN patches was only at the level of 16.1 ± 1.3% (n = 3) of the antigen content in the formulation employed for the MN patch fabrication (Appendix A, Figure A1). These results are consistent with previous observations on the protein-stabilizing effect of PCPP in aqueous solutions and coating processes [36,37,38].

### 3.5. Evaluation of Anti-GP Serological Responses by ELISA and Neutralization Assays

**Figure 5:**
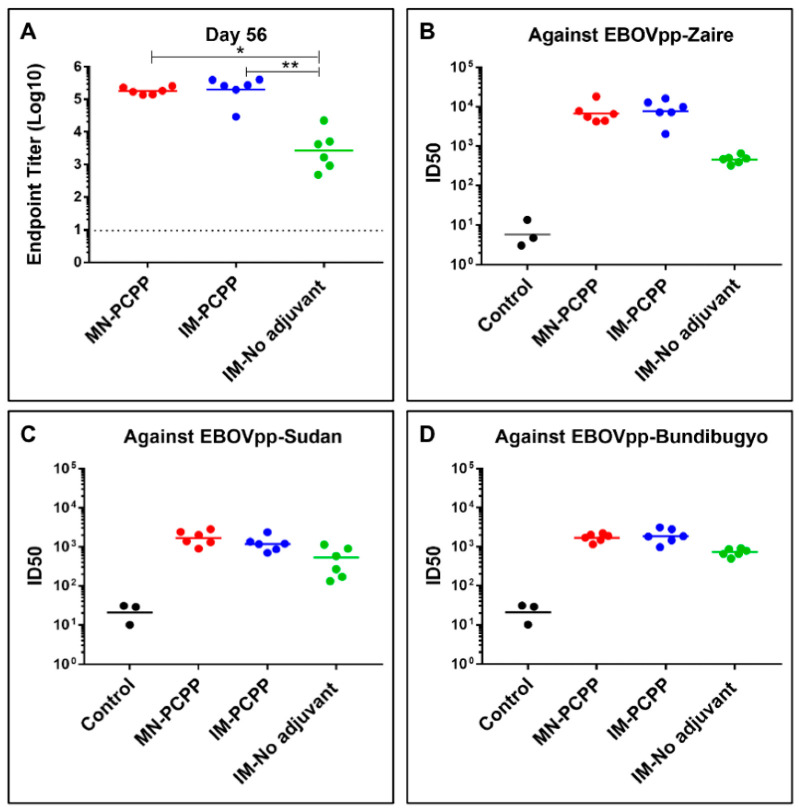
Analysis of antibody responses by microneedle patch (MN) or intramuscular (IM) delivery of EBOV GP with PCPP adjuvant or without adjuvant (No adjuvant). Mice (groups of 6) were vaccinated twice (Day 0 and Day 28) with 13.4 µg of EBOV GP. The control group represents pre-immune sera. Serum samples were collected at Day 56, i.e., 4 weeks after the second injection. (**A**) Endpoint titers were determined by ELISA using purified GP as the coating antigen. Neutralizing activity of sera was determined by incubating 10,000 RLU/mL of EBOVpp strains—(**B**) EBOVpp-Zaire, (**C**) EBOVpp-Sudan, and (**D**) EBOV GP Bunddibugyo—with serial two-fold dilutions of serum samples from each vaccinated mouse. Neutralization was measured as percentage decrease in luciferase expression compared with pre-immune sera controls, and plotted as ID50 values. *p* values for between-group endpoint titer values were calculated using Kruskal-Wallis analysis of variance with Dunn’s multiple-comparison test, and significant *p* values for comparisons of results between the immunization groups are shown (*, *p* ≤ 0.05; **, *p* ≤ 0.01).

The immune responses to the EBOV GP vaccine administered by dissolvable PCPP-based MN patches were investigated in comparison to IM injection using PCPP at the same dose. As shown in Figure 5, three groups of BALB/c mice, six mice per group, were immunized with GP formulated with MN-PCPP, IM-PCPP, and IM-No adjuvant as a no adjuvant control. After the first vaccination on Day 0, each group of mice received another vaccination on Day 28, and serum samples were collected on Days 0, 14, 28, 42, 56, 180, and 210 at the time of challenge. On Day 56, the endpoint titers between the MN-PCPP and IM-PCPP groups were not significantly different (Figure 5A). However, the endpoint titers induced by MN-PCPP and IM-PCPP were approximately 5-fold higher than IM-No adjuvant (based on Kruskal–Wallis analysis of variance with Dunn’s multiple comparison test).

To assess the ability of sera from the vaccinated mice to inhibit EBOV infection in vitro, serum samples were analyzed for their neutralizing activity against EBOV GP pseudoparticles from three isolates, EBOVpp-Zaire, homologous to the vaccine, EBOVpp-Sudan, and EBOVpp-Bundibugyo. As shown in Figure 5B, higher neutralization activities were detected against EBOVpp-Zaire in both GP administered by MN-PCPP and IM-PCPP versus the no adjuvant group (IM-No adjuvant). At a 1:400 dilution, sera from MN-PCPP and IM-PCPP immunized mice neutralized over 95% EBOV GP pseudoparticles and the ID50 titers were 10- to 100-fold higher than the IM-No adjuvant group. Interestingly, the breadth of neutralization against EBOVpp-Sudan and EBOVpp-Bundibugyo isolates was notable (Figure 5C,D) but with ID_50_ values 10-fold less than the than the homologous EBOV-Zaire isolate, which is perhaps indicative of the 40 percent sequence divergence of the isolates [56].

### 3.6. Kinetics and Durability of Antibody Responses and Protection against Lethal EBOV Challenge

**Figure 6:**
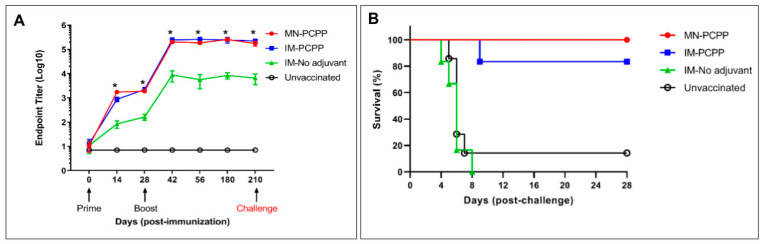
Kinetics and durability of antibody responses and protective efficacy against lethal mouse-adapted EBOV challenge. Mice (groups of 6) were vaccinated twice (Day 0 and Day 28) with 13.4 µg of EBOV GP by microneedle patch (MN) or intramuscular (IM) delivery of EBOV GP with PCPP adjuvant or without adjuvant (No adjuvant). Serum samples were taken from the mice over the course of 210 days until just prior to the challenge. The unvaccinated group represents a control group that did not receive vaccine. (**A**) Endpoint titers were determined by ELISA using purified GP as the coating antigen at the indicated timepoints. Statistically significant differences between the MN-PCPP and IM-No adjuvant groups are indicated by asterisk (*). (**B**) Daily survival rate of mice in each group post-challenge. Kaplan–Meier survival curve for mice in each group post-challenge. Statistical analysis using the log-rank test indicates that the survival curves exhibit statistically significant differences (*p* < 0.0001).

As shown in Figure 6A, the kinetics and durability of the anti-GP responses induced in both MN-PCPP and IM-PCPP-immunized mice were similar, with higher levels of antibody responses compared to the IM-No adjuvant group beginning at Day 14 (*p* < 0.05 based on Kruskal–Wallis analysis of variance with Dunn’s multiple comparisons test). When coating with 200 ng Zaire EBOV GPΔmuc per well for ELISA test, Zaire GP-specific antibody responses were detected as early as two weeks after the primary injection. The antibody levels against Zaire GP reached a peak two weeks after the second injection on Day 28, and the immune responses remained at a steady-state level through Day 210 at the time of challenge.

To compare the protective efficacy of the different immunization approaches, vaccinated mice were challenged with 1000 plaque-forming units (pfu) of mouse-adapted EBOV (MA-EBOV) via intraperitoneal injection on Day 210, more than six months after the second immunization on Day 28. The mice were monitored for disease symptoms and survival rates on a daily basis. As shown in Figure 6B, all mice in the MN-PCPP group survived to the terminal euthanasia on Day 28 with no findings suggestive of EBOV infection. Five of the six mice in the IM-PCPP group survived to the terminal euthanasia with no EBOV-related findings. In contrast, all six mice assigned to the IM-No adjuvant and five of six mice in the unvaccinated group died within 8 days post-challenge. Unscheduled-death mice (those that died before 28 days) had microscopic findings consistent with acute EBOV infection, including hepatocellular necrosis, inflammation, and intracytoplasmic inclusion bodies and splenic decreased lymphocytes and lymphocytolysis. In contrast to acutely fatal EBOV in macaques [57], fibrin deposition was an uncommon finding in affected mice, and when present, was limited in distribution and amount. This feature in mice has been previously reported [58].

An interesting microscopic finding, increased immunoblasts, was noted in unscheduled-death mice from the IM-PCPP, IM-No adjuvant, and unvaccinated groups (Group 3). The finding was commonly noted in the spleen but only noted in the liver in the single unscheduled-death mouse in the IM-PCPP group. The distribution of these large blast cells is similar to extramedullary hematopoiesis commonly noted in mice; however, the uniformity of the blast cell population with the absence of maturing erythroid, myeloid, or megakaryocytic lineages is distinct. Evaluation of bone marrow may assist in the identification and pathogenesis of these immature round cells, and immunohistochemical study would likely reveal a specific cell type.

Survival of one unvaccinated control group mouse as noted in this study has been reported previously in BALB/c mice exposed to murine-adapted EBOV [59]. The 100% survival of MN-PCPP group mice with absence of EBOV-related microscopic findings confirms the effectiveness of the MN patch technology in this study. Collectively, these results show that immunization with MN-PCPP can confer complete protection against a lethal challenge of EBOV, even after an extended period of time.

## 4. Conclusions

Currently, licensed EBOV vaccines all use viral vector platforms expressing the EBOV GP antigen. GP is located on the surface of the virion and serves as the main target of a neutralizing antibody response and protection against lethal EBOV challenge in mice, guinea pigs, and macaques [7,24,60,61,62]. However, these viral vaccine vectors have general disadvantages, such as reliance on expert administration by trained health care providers, making rapid deployment difficult in the event of an emergency. In view of this, our results address these limitations by showing we can fabricate a needle-free MN-adjuvant delivery system containing the GP antigen formulated with PCPP that can mount a durable immune response over 210 days and achieve complete protection against an Ebola virus challenge in a mouse model system. Such a simple-to-administer, patch-based delivery system may be used for simplified logistics of vaccination in low-resource and emergency settings.

This study demonstrated that the polyphosphazene immunoadjuvant, PCPP, played a central role in the induction of a robust immune response using skin immunization. Its immunoadjuvant potency with EBOV GPΔMuc, which was tested using the IM administration route, can be best illustrated by the results of the challenge study: 80% survival for animals immunized IM with adjuvanted formulations vs. 0% for the non-adjuvanted group. In the microfabrication process, this polymer-based material was compatible with the dissolvable MN patch technology. Furthermore, PCPP-based MN patches displayed over four-fold increase in the antigenicity compared to their CMC counterparts, essentially enabling EBOV GPΔMuc MN patch technology in the present study. As discussed above, this protein-stabilizing effect of PCPP may be correlated with the unique ability of this polymer to spontaneously form supramolecular assemblies with vaccine antigens. The importance and robustness of this feature of highly charged and flexible PCPP now find their validation in the field of solid-state formulations.

Future studies should look at the development of GP-based vaccines that confer protection against other filovirus species such as the Sudan, Bundibugyo, and Taï Forest viruses, including the Marburg virus genus. Further optimization of PCPP adjuvant and MN patch delivery platform, especially its shelf-life, will provide a means to develop a safe, effective, and rapidly deployable vaccine against EBOV and other filoviruses.

## Data Availability

Data are available upon reasonable request.
